# Statins and Histone Deacetylase Inhibitors Affect Lamin A/C – Histone Deacetylase 2 Interaction in Human Cells

**DOI:** 10.3389/fcell.2019.00006

**Published:** 2019-01-31

**Authors:** Elisabetta Mattioli, Davide Andrenacci, Antonello Mai, Sergio Valente, Joke Robijns, Winnok H. De Vos, Cristina Capanni, Giovanna Lattanzi

**Affiliations:** ^1^CNR Institute of Molecular Genetics, Unit of Bologna, Bologna, Italy; ^2^IRCCS Istituto Ortopedico Rizzoli, Bologna, Italy; ^3^Department of Drug Chemistry and Technologies, Pasteur Institute Italy, Cenci-Bolognetti Foundation, Sapienza University of Rome, Rome, Italy; ^4^Laboratory of Cell Biology and Histology, Department of Veterinary Sciences, University of Antwerp, Antwerp, Belgium

**Keywords:** prelamin A, *LMNA* gene, HDAC2, statins, HDAC inhibitors, trichostatin A (TSA), chromatin

## Abstract

We recently identified lamin A/C as a docking molecule for human histone deacetylase 2 (HDAC2) and showed involvement of HDAC2-lamin A/C complexes in the DNA damage response. We further showed that lamin A/C-HDAC2 interaction is altered in Hutchinson-Gilford Progeria syndrome and other progeroid laminopathies. Here, we show that both inhibitors of lamin A maturation and small molecules inhibiting HDAC activity affect lamin A/C interaction with HDAC2. While statins, which inhibit prelamin A processing, reduce protein interaction, HDAC inhibitors strengthen protein binding. Moreover, treatment with HDAC inhibitors restored the enfeebled lamin A/C-HDAC2 interaction observed in HGPS cells. Based on these results, we propose that prelamin A levels as well as HDAC2 activation status might influence the extent of HDAC2 recruitment to the lamin A/C-containing platform and contribute to modulate HDAC2 activity. Our study links prelamin A processing to HDAC2 regulation and provides new insights into the effect of statins and histone deacetylase inhibitors on lamin A/C functionality in normal and progeroid cells.

## Introduction

Histone-modifying enzymes are fine regulators of chromatin remodeling and include histone methyl-transferases and demethylases, histone acetyltransferases and deacetylases, histone kinases and ubiquitin ligases. Histone deacetylases (HDACs), which counteract histone acetylation favoring a repressive chromatin status, belong to three major classes and a fourth class that only includes HDAC11 (Seto and Yoshida, 2014). Class I HDACs, encompassing HDAC1, HDAC2, HDAC3, and HDAC8, are ubiquitously expressed and are mainly localized within the nucleus, where they deacetylate diverse histone residues to modulate transcription and other nuclear processes (Seto and Yoshida, 2014). In particular, class I HDACs are involved in DNA damage signaling and it has been reported that HDAC1 and -2 have a central role in preparing the chromatin for the activation of DNA damage response (DDR) (Roos and Krumm, 2016). Furthermore, it has been demonstrated that HDAC2 is involved in DDR through regulation of acetylation of H4K16 and H3K56 (Miller et al., 2010). Class II HDACs (HDAC4-7, -9 and -10) are expressed in a tissue-specific way and are mostly cytoplasmic, some of them have been reported abundant also in nucleus (HDAC6) (Seidel et al., 2015) and others translocate into the nucleus upon stimulus-induced phosphorylation and are exported to the cytoplasm upon binding to 14-3-3 protein (Nishino et al., 2008; Di Giorgio et al., 2015). Class III HDACs, known as sirtuins and including SIRT1-7, are located in the nucleus or in the cytoplasm and also in mitochondria and regulate acetylation of histones involved in transcriptional regulation, metabolism and DDR (Roos and Krumm, 2016). Furthermore, sirtuins are involved in aging pathways through acetylation of histones or non-histone substrates (Saunders and Verdin, 2007; Watroba and Szukiewicz, 2016). It has been reported that lamin A/C regulates sirtuin activity and defects of sirtuin anchorage (Cenni et al., 2014) and deacetylase function were observed in progeroid laminopathies (Ghosh et al., 2013; Liu and Zhou, 2013; Ghosh et al., 2015). Among progeroid laminopathies, Hutchinson-Gilford Progeria syndrome (HGPS) is a rare premature aging disease caused by mutations in *LMNA* gene and, in most cases, production of a truncated prelamin A form called progerin (Pellegrini et al., 2015). As in most laminopathies, chromatin dynamics are altered in HGPS and heterochromatin organization, histone methylation and acetylation and DDR are severely affected (Columbaro et al., 2005; Pellegrini et al., 2015; Evangelisti et al., 2016). We recently found that lamin A/C interacts with HDAC2 and influences HDAC2 recruitment to the p21 promoter, while lamin A/C-HDAC2 interaction is reduced in HGPS cells (Mattioli et al., 2018). We also observed that lamin A/C-HDAC2 interaction is decreased during DDR and recovered at completion of DNA repair in control human fibroblasts, whereas this modulation is lost in HGPS cells (Mattioli et al., 2018). Furthermore, we showed that lamin A/C interacts with HDAC2 to promote its deacetylase activity and also this function is altered in HGPS cells (Mattioli et al., 2018).

HDACs can be inhibited by an increasing number of inhibitors, among which the best known is trichostatin A (TSA), a potential therapeutic compound for cancer and many other diseases (Seto and Yoshida, 2014). This is because TSA (and by extension other HDAC inhibitors) may be used to impair DDR and favor cell death, as in oncological applications, or rather to activate transcription of repressed sequences, as in muscular dystrophies (Bajanca and Vandel, 2017). We previously demonstrated that the combined inhibition of HDAC activity (with TSA) and prelamin A/progerin farnesylation (with mevinolin) rescues aberrant chromatin organization and transcriptional activity in cells from HGPS (Columbaro et al., 2005). Those cells accumulate progerin, a truncated and farnesylated form of the lamin A precursor (Mattioli et al., 2018). The lamin A precursor, also known as prelamin A, is produced as the main splicing product of the *LMNA* gene and undergoes a complex post-translational processing leading to lamin A maturation. The C-terminal CSIM sequence of prelamin A undergoes farnesylation, cleavage by the Zinc-dependent metalloprotease Zmpste24 and carboxymethylation. Thereafter, removal of the last 15 amino acids through a second Zmpste24-mediated cleavage yields mature lamin A (Worman and Michaelis, 2018). Statins inhibit the HMG-CoA reductase activity, which is necessary for production of the farnesyl group (Mattioli et al., 2008). As farnesylation is the first modification of prelamin A and it is required for further post-translational processing, statins cause accumulation of unprocessed, non-farnesylated prelamin A in cells (Bikkul et al., 2018). Here we show that HDAC inhibitors as well as inhibitors of lamin A maturation modulate the interaction of lamin A/C with HDAC2. In particular, while statins reduce, HDAC inhibitors strengthen protein interaction. Importantly, TSA rescues lamin A/C-HDAC2 interaction in HGPS cells. Based on these results, we propose that prelamin A levels as well as HDAC2 activation status influence the extent of HDAC2 recruitment to A-type lamins and contribute to modulate HDAC2 activity. These results increase our understanding of the effect of drugs on lamin-dependent epigenetic mechanisms and support the hypothesis that TSA could represent a therapeutic tool for HGPS.

## Materials and Methods

### Cell Culture and Transfection

Skin fibroblast cultures were obtained from skin biopsies of healthy subjects undergoing orthopedic surgery due to trauma or laminopathic patients, following a written consent. HGPS fibroblasts carrying heterozygous p.G608G *LMNA* mutation were from two patients aged 3 and 5 years. All cell cultures were from the BioLaM biobank. The experimental protocol had been approved by the local ethical committee (Rizzoli Orthopedic Institute Ethical Committee approval Prot. Gen. 0018250 – 2016) and followed EU rules. Cell cultures were established and cultured in Dulbecco’s modified Eagle’s medium, supplemented with 20% fetal bovine serum (FBS) from Gibco Life Technology and antibiotic-anti-mycotic solution from Sigma. HEK 293 and HeLa cells were cultured in Dulbecco’s modified Eagle’s medium, supplemented with 10% FBS. The HeLa *LMNA* knockout (*LMNA*-/-) cell lines were generated using CRISPR-Cas9 mediated genome editing technology with a guide RNA targeting the first exon of the *LMNA* gene (5′-CCTTCGCATCACCGAGTCTGAAG-3′), as described before (Robijns et al., Scientific Reports 2016; Houthaeve et al., ACS Nano 2018). Constructs containing the Cas9 nuclease and selection markers were obtained from Addgene (#48138 and 48139) and published protocols were followed (Ran et al., Nature Protocols 2013). Control cells (*LMNA* +/+) underwent the same treatment with a construct containing no guide RNA.

### Plasmids

HEK293 cells were transfected with FLAG-tagged plasmids containing: wild-type prelamin A, LA-WT, which undergoes normal maturation; LA-C661M, which cannot be farnesylated; LA-L647R, which is farnesylated but cannot undergo endoproteolysis and delta50 construct that mimic HGPS patient (Lattanzi et al., 2007). Transfection of HEK293 cells was performed using Fugene 6 solution (Promega) according to the manufacturer’s instructions and cells were incubated for 24 h after transfection.

### Antibodies and Drugs

Antibodies employed were: anti-lamin A/C, goat polyclonal [Santa Cruz, SC-6215, used at 1:100 dilution for WB and *in situ* proximity ligation assay (PLA)]; anti lamin A/C (Novocastra, used for IP); anti-prelamin A, goat polyclonal (Santa Cruz, SC-6214, used 1:100 for WB and for PLA analysis); 1188-1 rabbit-polyclonal anti-prelamin A (non-farnesylated) (Diatheva used 1:50 for IF); 1188-2 rabbit-polyclonal anti-prelamin A (farnesylated) (Diatheva used 1:10 for IF); anti-HDAC2, rabbit polyclonal (Abcam, AB16032, used 1:3000 for WB and 1:200 for PLA analysis); anti-HDAC2, mouse monoclonal (Santa Cruz, SC-55541 used 1:100 for PLA analysis); anti-H4K16 acetylated, rabbit polyclonal (Abcam used 1:4000 for WB and 1:200 for PLA analysis); anti-H3K9 acetylated, rabbit polyclonal (Abcam, used 1:500 for WB and 1:200 for PLA); anti-Flag tag (Sigma, 1:3000 for WB); anti-53BP1 (for IF); anti-GAPDH (Millipore 1:5000 for WB).

The HDAC inhibitor TSA (Sigma) was applied 1.5 μM for 18 h to cell cultures to induce Class I/II HDAC inhibition. MS-275 (entinostat), a synthetic benzamide inhibitor selective for HDAC1-3, was applied 5 μM for 18 h. MC1568, a class II HDAC/HDAC8 inhibitor (Panella et al., 2016) was applied 5 μM for 18 h. Mevinolin (Sigma) was added 25 μM to human fibroblasts and 3 μM to HeLa cells for 18 h. N-acetyl-S-farnesyl-L-cysteine methylester (AFCMe, from Alexis), a non-peptidomimetic ZMPSTE24 inhibitor, was added (10 μM) to cell cultures for 18 h to accumulate farnesylated prelamin A (Cenni et al., 2011). Mevinolin causes non-farnesylated prelamin A accumulation through inhibition of farnesyl production. AFCMe is a non-peptidomimetic small molecule that impairs binding of the prelamin A endoprotease Zmpste24, thus impairing cleavage and maturation of farnesylated prelamin A (Mattioli et al., 2008). The dosage of mevinolin and TSA for combined treatment was previously reported (Columbaro et al., 2005). To induce oxidative stress, 100 μM hydrogen peroxide (H_2_O_2_) was added to human dermal fibroblast cultures for 4 h. Recovery was measured 48 h after H_2_O_2_ removal (Mattioli et al., 2018).

### *In situ* Proximity Ligation Assay

The PLA experiments were performed using kits from Sigma-Aldrich: Duolink^®^ *in situ* Detection Reagents Orange (DUO92007) according to manufacturer instructions. Briefly, methanol-fixed samples were treated with 4% BSA in PBS to saturate non-specific binding and incubated with primary antibodies overnight at 4°C. Thereafter, slides were incubated for 1 h at 37°C with secondary probes diluted to final concentrations of 1:5. Ligation solution was added to each sample and slides were incubated in a humidity chamber for 30 min at 37°C. Later ligation solution was removed with wash buffer A and amplification solution was added to each sample. Slides were incubated in a humidity chamber for 100 min at 37°C and then washed with wash buffers. Duolink *in situ* mounting medium with DAPI was added to the slides and samples were observed by a Nikon Eclipse Ni fluorescence microscope equipped with a digital CCD camera and NIS Elements AR 4.3 software. Quantitative analysis of PLA results was performed using Duolink Image Tool software (Sigma) by counting 200 nuclei per sample.

### Immunofluorescence Analysis

Cells grown on coverslips were fixed with absolute methanol at -20°C for 7 min. After saturation of non-specific binding with PBS containing 4% BSA, coverslips were incubated with primary antibodies overnight at 4°C or 1 h, and revealed with FITC or TRIC-conjugated secondary antibodies diluted 1:100 (incubated for 1 h at RT). Samples were mounted with an anti-fade reagent (Molecular Probes, ThermoFisher) and observed with a Nikon Eclipse Ni epifluorescence microscope. The images captured with NIS- Elements 4.3 AR software were processed using Photoshop CS.

### Immunoblotting and Immunoprecipitation

For Western blot analysis, human fibroblasts were lysed in a buffer containing: 20 mM TRIS–HCl (*pH* = 7.5), 1% SDS, 1 mM Na_3_VO_4_, 1 mM PMSF, 5% beta-mercaptoethanol and protease inhibitors. After sonication centrifugation and protein quantification by Bradford method, proteins were subjected to SDS gradient gel (5–20%) electrophoresis and transferred to nitrocellulose membrane overnight at 4°C. Incubation with primary and secondary antibodies was performed and immunoblotted bands were revealed by Invitrogen ECL detection system.

For Immunoprecipitation experiments (IP) transfected HEK293 cells or HeLa cells were lysed in high detergent-IP buffer containing: 50 mM TRIS–HCl (*pH* = 8), 250 mM NaCl, 0.1% SDS, 1% NP-40, 1 mM PMSF, and protease and phosphatase inhibitors. For each samples 700 μg of lysate were incubated over-night with 1 μg of anti-FLAG tag from Sigma or 12 μl of anti-lamin A/C form Novocastra or non-specific immunoglobulins form Santa Cruz Biotechnology as a negative control. After the addition of 30 μl of protein A/G (Santa Cruz Biotechnology) for 60 min at 4°C, the immunoprecipitated proteins were washed three times in IP buffer. Later the samples were added to Laemmli’s buffer, boiled and subjected to western blot analysis. The intensity of bands was measured using a BioRad GS800 Densitometer.

### Statistical Analysis

Graphs in each panel represent mean values from at least three independent experiments +/- standard error. Statistically significant differences (*p* < 0.05) are calculated by Student’s *t*-test.

## Results

### Modulation of Lamin A/C-HDAC2 Interaction by Statins

**Figure 1:**
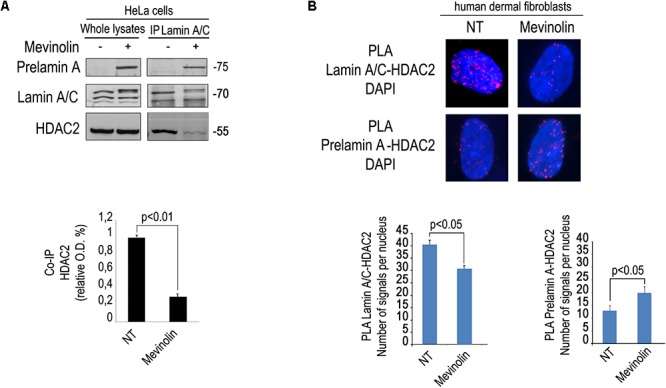
Modulation of lamin A/C-HDAC2 interaction by statins. **(A)** Co-immunoprecipitation of lamin A/C and HDAC2 in the presence or absence of mevinolin as indicated in the legend. A representative western blot is shown. Molecular weight markers are indicated in kDa. Densitometric analysis of immunoprecipitated HDAC2 bands is reported in the graph. Statistical significance (*p* < 0.01) is indicated. **(B)** PLA of lamin A/C and HDAC2 or prelamin A and HDAC2 in human dermal fibroblasts left untreated (NT) or treated with mevinolin (Mevinolin). Nuclei are counterstained with DAPI. Quantitative analysis of PLA signals (red dots) is reported in the graphs. Statistical significance (*p* < 0.05) is indicated.

We first tested the effect of mevinolin, a drug affecting prelamin A processing, on the interaction between HDAC2 and lamin A/C using an antibody that recognizes all forms of A-type lamins including the precursors. To this end, we performed co-immunoprecipitation experiments in cells treated with mevinolin that, as all statins, impairs HMG-CoA reductase activity and farnesyl production inducing non-farnesylated prelamin A accumulation (Mattioli et al., 2008; Lattanzi et al., 2014). Moreover, we measured lamin A/C-HDAC2 complexes in fibroblasts that had accumulated farnesylated prelamin A due to AFCMe treatment (Mattioli et al., 2008; Lattanzi et al., 2014). Lamin A/C-HDAC2 complexes were detected in untreated cells, whereas a lower amount of HDAC2 co-immunoprecipitated in mevinolin treated cells (Figure 1A). PLA confirmed reduced lamin A/C-HDAC2 interplay in mevinolin-treated skin fibroblasts (Figure 1B). However, the interaction of lamin A/C with HDAC2 in cells that had accumulated farnesylated prelamin A was comparable to that observed in untreated cells (Supplementary Figure S1). Thus, mevinolin reduces lamin A/C affinity for HDAC2. Based on these data, we suspected that accumulation of non-farnesylated prelamin A could reduce lamin A/C-HDAC2 interaction.

### Low Affinity of Non-farnesylated Prelamin A for HDAC2 Interaction

**Figure 2:**
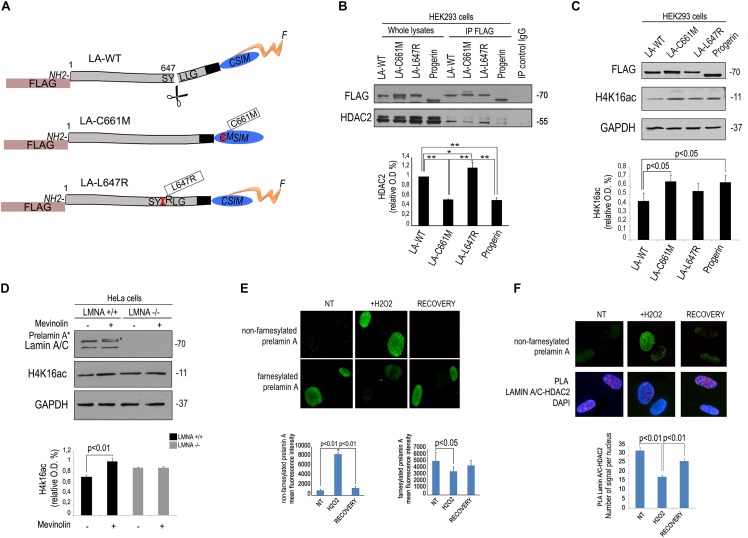
Low affinity of HDAC2 for non-farnesylated prelamin A. **(A)** Schematic representation of lamin A mutants used in these experiments. The mutation is reported in boxes. The name of each plasmid (LA-WT, LA-C661M, LA-L647R), the tag (FLAG) and mutations (crossed amino acid replaced by the following) are indicated; F, farnesyl residue. **(B)** Co-immunoprecipitation of FLAG-lamin A and endogenous HDAC2 in HEK293 cells expressing FLAG-tagged wild-type lamin A (LA-WT), non-farnesylable prelamin A (LA-C661M), uncleavable farnesylated prelamin A (LA-L647R) and lamin A delta 50 encoding progerin (progerin). Quantitative analysis of HDAC2 IP band normalized on immmoprecipitated FLAG was performed. Statistical significance is indicated by asterisks (^∗∗^*p* < 0.01; ^∗^*p* < 0.05). **(C)** Western blot analysis of H4K16ac in human HEK293 cells expressing FLAG-tagged wild-type lamin A (LA-WT), non-farnesylable prelamin A (LA-C661M), uncleavable farnesylated prelamin A (LA-L647R) and lamin A delta 50 encoding progerin (progerin). GAPDH bands are shown as loading control. Quantitative analysis of HDAC2 IP band normalized on immmoprecipitated lamin A/C was performed. **(D)** Western blotting analysis of lamin A/C and H4K16ac in *LMNA* +/+ and *LMNA* -/- in absence or presence of mevinolin. Densitometric analysis of H4K16ac bands is reported in the graph. GAPDH was used as a loading control. **(E)** Immunofluorescence staining of non-farnesylated prelamin A and farnesylated prelamin A during oxidative stress-induced DDR. Nuclei from untreated human fibroblasts (NT), cells after 4 h H2O2 treatment (H2O2) or after H2O2 treatment and 48 h recovery (RECOVERY) are shown. Quantitative analysis of mean fluorescence intensity measured in 100 nuclei from each sample is shown in the graphs. Statistical significance (*p* < 0.01 or *p* < 0.05) is indicated. **(F)** Immunofluorescence analysis of prelamin A and PLA of lamin A/C and HDAC2 in the same samples shown in **(D)**. Nuclei are counterstained with DAPI. Quantitative analysis of PLA signals is shown in the graph. Statistical significance (*p* < 0.01) is indicated.

As a first step towards the understanding of mevinolin effects on lamin A/C-HDAC2 interaction, we transfected FLAG-tagged lamin A or unprocessable prelamin A mutants (Figure 2A) in human HEK293 cells and co-immunoprecipitated HDAC2 using a FLAG-directed antibody. Both prelamin A forms (LA-C661M, non-farnesylated prelamin A and LA-L647R, farnesylated prelamin A) and mature lamin A co-precipitated HDAC2 (Figure 2B). However, non-farnesylated prelamin A showed low affinity for HDAC2 (Figure 2B). Also progerin, the prelamin A form found in HGPS, showed reduced interaction with HDAC2 relative to other *LMNA* products (Figure 2B). Since progerin is a truncated and farnesylated prelamin A form, our observation suggested that the amino acid sequence deleted in progerin could be involved in HDAC2 interaction.

Of note, in cells expressing non-farnesylated prelamin A or progerin, acetylation of the HDAC2 substrate H4K16 was slightly, but significantly increased (Figure 2C), supporting previous observations linking lamin A/C-HDAC2 interaction to enzyme activity (Mattioli et al., 2018). Moreover, we observed that in cells where *LMNA* expression had been knocked-out by CRISPR/Cas9 technology, mevinolin treatment did not affect acetylation of H4K16 (Figure 2D). This result strongly indicated that mevinolin-induced changes of H4K16 acetylation levels are due to accumulation of non-farnesylated prelamin A and altered lamin A/C-HDAC2 interaction.

We previously reported that lamin A maturation is slowed down and prelamin A is accumulated at the early stages of DDR, while its processing rate is increased and precursors almost undetectable upon DNA damage recovery (Lattanzi et al., 2014). Here, we confirmed that diverse prelamin A forms were accumulated at different stages during stress response. Increase of non-farnesylated prelamin A was observed after 4 h of oxidative stress, while this form of the lamin A precursor was undetectable upon DNA damage recovery (Figure 2E). In cells subjected to oxidative stress, we could also detect prelamin A-HDAC2 complexes (Figure 2F). Moreover, an inverse correlation was determined between levels of non-farnesylated prelamin A and lamin A/C-HDAC2 interaction during DDR (Figure 2F). The latter finding, suggested that prelamin A increase contributed to the observed reduction of lamin A/C-HDAC2 interaction during DDR (Mattioli et al., 2018).

### Modulation of Lamin A/C –HDAC2 Interaction by HDAC Inhibitors

**Figure 3:**
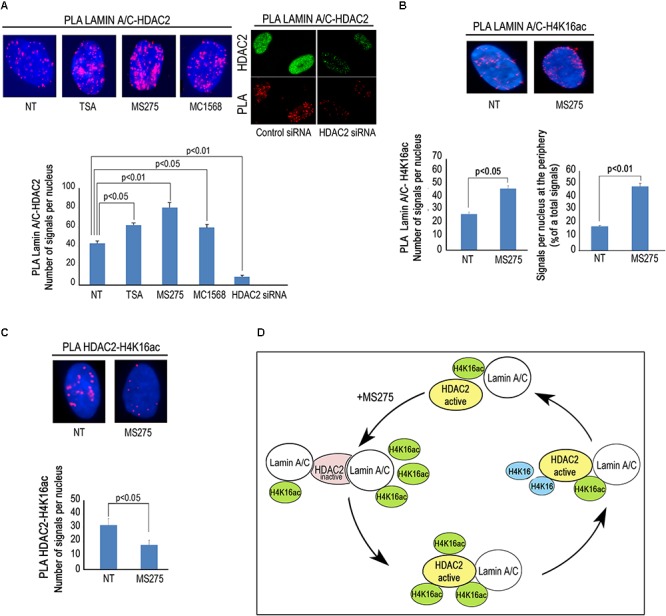
HDACs inhibitors influence lamin A/C-HDAC2 interaction. **(A)** PLA of lamin A/C and HDAC2 in human dermal fibroblasts left untreated (NT) or treated with HDAC inhibitors TSA (TSA), MS-275 (MS275) or MC1568 (MC1568). PLA of lamin A/C and HDAC2 in cells subjected to HDAC2 knockdown (siRNA HDAC2) or to a control siRNA (control siRNA) is shown in the right panel. HDAC2 was labeled by a specific antibody to show protein downregulation (HDAC2). Red dots, PLA signals. Quantitative analysis of PLA signals is reported in the graph. **(B)** PLA of lamin A/C and H4K16ac in human fibroblasts left untreated or treated with MS-275. Quantitative analysis of total PLA signals and percentage of signals at the periphery with respect to total signals are indicated in the graphs. **(C)** PLA of HDAC2 and H4K16ac in fibroblasts left untreated or treated with MS 275. Quantitative analysis of PLA signals is reported in the graph. **(D)** Cartoon representing a working hypothesis based on the assumption that active HDAC2 (yellow), upon inhibition by MS-275, changes its conformation (pink) thus increasing its affinity for lamin A/C. Lamin A/C binds H4K16ac and inactive HDAC2, lamin A/C binding activates HDAC2 and de-acetylated H4K16 is released. Nuclei in **(A**–**C)** are counterstained with DAPI. Statistical significance (*p* < 0.05 or *p* < 0.01) is indicated.

Then, we wished to test if modulation of HDAC activity by HDAC inhibitors could affect lamin A/C–HDAC2 interaction. To this purpose, we treated human dermal fibroblasts with TSA, a well-known pan-HDAC inhibitor, MS-275, a class I-selective HDAC inhibitor active against HDAC1, HDAC2, and HDAC3, and MC1568 to be used as a negative control. Unexpectedly, all treatments increased HDAC2 recruitment by lamin A/C (Figure 3A). The increased interaction after addition of TSA and MS-275 suggests that the catalytically inactive conformation of the enzyme has a higher affinity for lamin A/C (Figure 3A). On the other hand, the effect observed with MC1568, inactive against HDAC2, could be in part explained by indirect posttranslational modification of HDAC2 elicited by the inhibitor. Indeed, HDAC2 has been reported to be acetylated by the histone acetyltransferase PCAF and deacetylated by HDAC5 (Eom et al., 2014). MC1568, an HDAC5 inhibitor, could increase HDAC2 acetylation level thus favoring lamin A/C interaction. It has been demonstrated that also TSA treatment increases HDAC2 acetylation (Eom et al., 2014). Based on these considerations, we speculate that HDAC2 acetylation could itself increase the interaction of lamin A/C with the enzyme. Treatment of fibroblasts with the HDAC2 inhibitor MS-275 also increased lamin A/C-H4K16Ac interaction, as determined by PLA (Figure 3B), possibly due to increased availability of the acetylated histone. Lamin A/C-H4K16Ac complexes were clearly detectable at the nuclear rim in more than 40% of quantified nuclei (Figure 3B), while, as expected, HDAC2-H4K16Ac binding decreased after MS-275 treatment (Figure 3C). In this scenario, we propose a model of lamin A/C-HDAC2 interaction based on the hypothesis that, in the presence of HDAC inhibitors, lamina A/C recruits histone substrates of HDAC2 (including H4K16Ac) to the inactive enzyme and favors its activity (Figure 3D).

### Rescue of Lamin A/C-HDAC2 Interaction in HGPS Cells by HDAC Inhibitors

**Figure 4:**
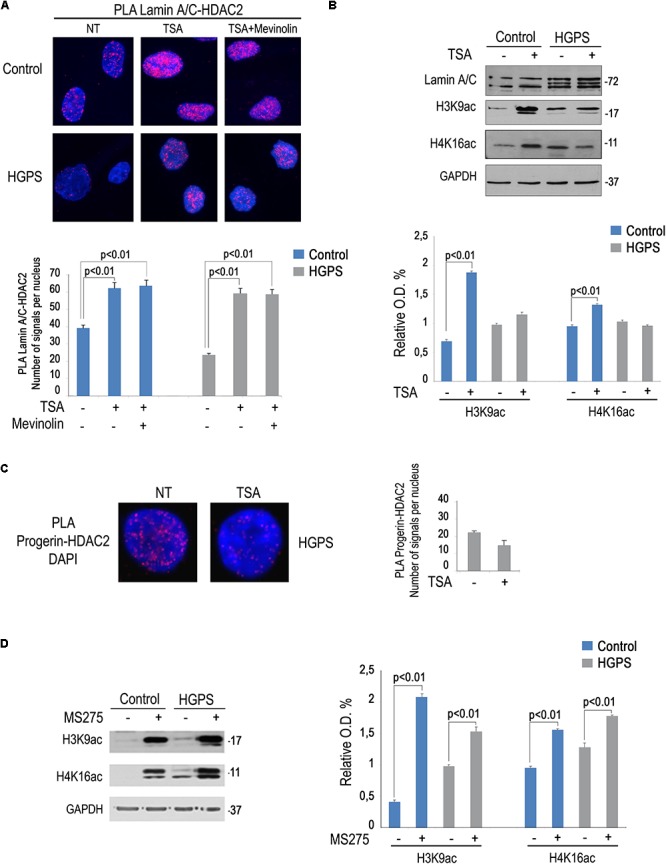
Rescue of lamin A/C-HDAC2 interaction in HGPS by TSA. **(A)** PLA of lamin A/C and HDAC2 in human dermal fibroblasts left untreated (NT), treated with TSA (TSA) or a combination of TSA and mevinolin (TSA + mevinolin). Quantitative analysis of PLA signals is reported in the graph. **(B)** Western blotting analysis of lamin A/C, H4K16ac and H3K9ac in control and HGPS fibroblasts left untreated or treated with TSA. GAPDH was used as a loading control. Densitometric analysis is reported in the graph. **(C)** PLA of progerin and HDAC2 in human dermal fibroblasts left untreated or treated with TSA. Quantitative analysis of PLA signals is reported in the graph. **(D)** Western blotting analysis of H4K16ac and H3K9ac in control and HGPS fibroblasts left untreated or treated with MS-275. GAPDH was used as a loading control. Densitometric analysis is reported in the graph. Nuclei in **(A**,**C)** are counterstained with DAPI. Statistical significance (*p* < 0.01) is indicated.

We previously reported that a combination of mevinolin and TSA is able to rescue several hallmarks of the HGPS cellular phenotype including peripheral heterochromatin loss, reduced transcriptional activity and altered nuclear morphology (Columbaro et al., 2005). The same drug combination was also effective in A type Mandibuloacral dysplasia cells (Camozzi et al., 2012). We recently discovered a reduced lamin A/C-HDAC2 interaction in HGPS cells (Mattioli et al., 2018). Here, we wanted to test TSA in HGPS fibroblasts and evaluate HDAC2-lamin A/C interaction and H4K16 acetylation levels. Increased lamin A/C-HDAC2 interaction was determined upon TSA treatment both in control and HGPS fibroblasts (Figure 4A). Moreover, the combined treatment with mevinolin and TSA also increased lamin A/C-HDAC2 interaction both in control and HGPS cells (Figure 4A). Of note, while in control fibroblasts TSA induced histone acetylation, as expected, this was not the case in HGPS (Figure 4B). In fact, while increased H3K9 and H4K16 acetylation were found in untreated HGPS cells, as reported before (Mattioli et al., 2018), H4K16ac and H3K9ac levels did not change after TSA treatment (Figure 4B). Moreover, we noticed that, while progerin (Figure 4B) or HDAC2 levels were not affected by TSA, this drug treatment reduced progerin–HDAC2 interaction (Figure 4C). This effect could contribute to rescue lamin A/C binding to HDAC2 in HGPS cells (Mattioli et al., 2018). This surprising result prompted us to test the effect of MS-275 that specifically inhibits class I HDACs including HDAC1 and HDAC2. MS-275 treatment also increased lamin A/C-HDAC2 interaction (Figure 3A), but also HDAC2 substrates H3K9 and H4K16 acetylation levels (Figure 4D). As a whole, these results indicate TSA as a good pharmacological tool for progeria cells as rescue of HDAC2-lamin A/C interaction occurs in TSA-treated HGPS cells, while excess histone acetylation is avoided.

## Discussion

In this paper, we show that mevinolin and HDAC inhibitors affect lamin A/C-HDAC2 interaction in human cells. Statins are known inhibitors of prelamin A processing leading to accumulation of non- farnesylated prelamin A (Columbaro et al., 2005). Their effect in HGPS (Columbaro et al., 2005; Varela et al., 2008; Harhouri et al., 2018) and that of other drugs able to accumulate non-farnesylated prelamin A (Gabriel et al., 2017) has been widely tested and shown to improve the cellular phenotype as well as disease symptoms in animal models. Some beneficial effect of inhibitors of prelamin A farnesylation have been also reported in clinical trials in HGPS patients (Gordon et al., 2018). However, the molecular target(s) downstream of non-farnesylated prelamin A in HGPS cells had been elusive. Here, we identify the lamin A/C-HDAC2 containing platform as a target of statins both in control and progeria cells and show that statins weaken lamin A/C-HDAC2 interaction. Importantly, the effect on the lamin A/C containing platform elicits acetylation of HDAC2 substrate H4K16, which supports the view that loss of lamin A/C interaction reduces HDAC2 activity (Mattioli et al., 2018). Although mevinolin is known to impair farnesylation of several molecules and cholesterol synthesis, the observation that this drug treatment does not elicit H4K16 acetylation in cells devoid of lamin A/C hints in favor of a direct role of prelamin A accumulation in mevinolin effect on HDAC2. Further, data obtained in cells overexpressing prelamin A forms, support the hypothesis that prelamin A processing rate influences lamin A/C interaction with HDAC2. In particular, non-farnesylated prelamin A, either accumulated by mevinolin treatment or by expression of a non-farnesylable prelamin A mutant, showed reduced affinity for HDAC2 and its substrate H4K16ac. Hence, in the presence of non-farnesylated prelamin A, reduced formation of lamin A/C-HDAC2 complexes is observed. However, farnesylated prelamin A accumulation does not interfere with lamin A/C-HDAC2 interaction. These findings are also relevant to the understanding of lamin A/C-HDAC2 interplay during DDR. In fact, we show that non-farnesylated prelamin A is accumulated during DDR. Thus, reduced formation of lamin A/C-HDAC2 complexes in cells subjected to oxidative stress could be explained by transient accumulation of the low affinity non-farnesylated form of the lamin A precursor. Transient increase of prelamin A levels during DDR had been previously reported and we had further shown that only cells from centenarians retain low amounts of farnesylated prelamin A at recovery from DNA damage, while prelamin A is almost undetectable in human fibroblasts under basal conditions (Lattanzi et al., 2014). In that study, we showed that 53BP1 import in nuclei upon oxidative stress induction was favored by prelamin A accumulation (Lattanzi et al., 2014). Data here reported, by showing that non-farnesylated prelamin A contributes to reduced HDAC2 activity and histone deacetylation, add further knowledge to the biological significance of transient prelamin A accumulation during DDR.

The different affinity of prelamin A forms for HDAC2 could play a role also in the pathogenesis of progeroid laminopathies, where accumulation of prelamin A occurs. In agreement with this hypothesis, we show that lamin A/C-HDAC2 binding is severely affected in cells from HGPS and other progeroid laminopathy patients [this paper and (Mattioli et al., 2018)]. In fact, the presence of progerin severely reduces lamin A/C-HDAC2 interaction. However, since progerin is a farnesylated form of prelamin A, and we show farnesylated prelamin A not to alter HDAC2 binding, we conclude that the 50 amino acids deleted in progerin are necessary to stabilize HDAC2-lamin A/C complexes. On the other hand, we previously showed that both non-farnesylated and farnesylated prelamin A are recovered in MADA cells (Camozzi et al., 2012). Thus, accumulation of non-farnesylated prelamin A could in part explain reduced lamin A/C-HDAC2 complexes in MADA (Mattioli et al., 2018).

In this paper, we show that lamin A/C-HDAC2 interaction can be also modulated by HDAC inhibitors. Strengthening of lamin A/C-HDAC2 binding by different HDAC inhibitors could be explained by conformational modifications of HDAC2 able to favor its interaction with lamin A/C. In particular, treatment with the HDAC1/2 inhibitor MS-275 was able to reduce the binding between HDAC2 and its substrate H4K16ac and increased lamin A/C- H4K16ac interaction, suggesting that lamin A/C could have higher affinity for the inactive enzyme. An hypothesis based on the assumption that lamin A/C targets acetylated H4K16 to HDAC2 and favors histone deacetylation is depicted in Figure 3D.

Further, our results support the hypothesis that the acetylation status of HDAC2 favors its binding with lamin A/C. In fact both TSA and the class II inhibitor MC1568, which trigger HDAC2 acetylation (Eom et al., 2014), increased lamin A/C-HDAC2 interaction. Since phosphomimetic or non-phosphorylable HDAC2 serine 394 mutants showed the same affinity for lamin A binding as the unmodified enzyme (Mattioli et al., 2018), we conclude that HDAC2 acetylation rather than phosphorylation, might regulate its binding properties relative to A type lamins. We suggest that lamin A/C preferentially recruits the inactive acetylated enzyme to trigger its activation. The finding that HDAC2 acetylation precedes HDAC2 phosphorylation on serine 394 supports this hypothesis (Eom and Kook, 2015). However, given the effect observed using diverse deacetylase inhibitors, we cannot rule out the possibility that even lamin A/C acetylation status could influence its binding to HDAC2. This aspect deserves further investigation.

Epigenetic changes and repeated oxidative stress induce the aging process (Guillaumet-Adkins et al., 2017). Indeed, a constitutively acetylated status of H4K16 induces a shortening of lifespan (Dang et al., 2009). We recently described a protein platform including HDAC2, H4K16ac, and lamin A/C, able to regulate epigenetic modifications of chromatin during DNA repair. Defects of lamin A/C within this platform could contribute to a defective DDR and induce cellular senescence. A reduced binding between lamin A/C and HDAC2 was in fact observed in HGPS cells (Mattioli et al., 2018). Furthermore, HGPS cells showed defective modulation of HDAC2 substrates, H3K9ac and H4K16ac, during oxidative stress response (Mattioli et al., 2018). In this paper, we show that TSA treatment of HGPS cells restores HDAC2-lamin A/C interaction and the effect is also obtained by combining mevinolin and TSA. These findings can in part explain the beneficial effect observed in HGPS fibroblasts following TSA treatment in combination with mevinolin (Columbaro et al., 2005). Importantly, TSA treatment elicited far different effects on histone acetylation in HGPS cells with respect to controls. In fact, very low, not statistically significant, increase of H4K16 and H3K9 acetylation was observed in HGPS cells subjected to TSA. This result could be explained by rescue of HDAC2 function in HGPS cells, due to recovery of HDAC2-lamin A/C interaction after TSA treatment. Considering that TSA binding to HDACs is a reversible process (Seto and Yoshida, 2014), rescue of HDAC2-lamin A/C binding could overcome the inhibitory effect of TSA and help restoring enzyme activity. This is particularly relevant for cellular senescence, as we recently showed that lamin A/C-HDAC2 interaction is related to expression of the senescence marker p21. In fact, not only loss of lamin A/C interaction, but also reduced binding of HDAC2 to the p21 promoter occurs in HGPS cells (Mattioli et al., 2018).

Thus, modulation of lamin A/C-HDAC2 interaction by HDACs inhibitors suggests a potential therapeutic approach in progeroid laminopathies, which warrants further investigation.

## Ethics Statement

This study was carried out in accordance with the recommendations of ‘Italian, local and EU rules’ with written informed consent from all subjects. All subjects gave written informed consent in accordance with the Declaration of Helsinki. The protocol was approved by the ‘Rizzoli Orthopedic Institute ethical committee’ in 2016 for the BioLaM project.

## Author Contributions

EM designed the study and performed all the PLA, immunofluorescence, and western blotting the experiments. DA performed all the statistical evaluation of results. SV and AM designed HDAC inhibitor studies and prepared reagents. JR produced *LMNA* knockout cells by CRISPR/Cas9 technology. WD produced *LMNA* knockout cells by CRISPR/Cas9 technology and critically reviewed the manuscript. CC performed the co-immunoprecipitation experiments and overexpression studies. GL designed the study, interpreted the results, and prepared the manuscript.

## Conflict of Interest Statement

The authors declare that the research was conducted in the absence of any commercial or financial relationships that could be construed as a potential conflict of interest.
