# Supplementary material for: Statins and Histone Deacetylase Inhibitors Affect Lamin A/C – Histone Deacetylase 2 Interaction in Human Cells
Source: Front Cell Dev Biol. 2019 Jan 31;7:6. doi: 10.3389/fcell.2019.00006 (PMC6365888; doi:10.3389/fcell.2019.00006)
Supplement: Supplementary file 1 [file Data_Sheet_1.pdf]

Supplementary Material

Statins and histone deacetylase inhibitors affect lamin A/C - histone deacetylase 2 interaction in human cells

Elisabetta Mattioli[1,2], Davide Andrenacci[1,2], Antonello Mai[3], Sergio Valente[3], Joke Robijns[4], Winnok H. De Vos[4], Cristina Capanni[1,2] and Giovanna Lattanzi[1,2*]

*** Correspondence:** Corresponding Author: giovanna.lattanzi@cnr.it

1.1 Supplementary Figures

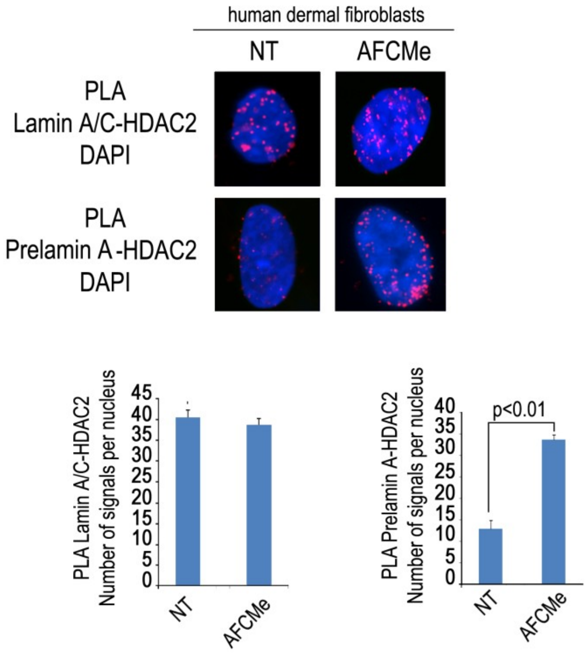

Supplementary figure 1. AFCMe induces accumulation of farnesylated prelamin A, but does not affect lamin A/C-HDAC2 interaction. PLA of lamin A/C and HDAC2 or prelamin A and HDAC2 in human dermal fibroblasts left untreated (NT) or treated with AFCMe (AFCMe). Nuclei are counterstained with DAPI. Quantitative analysis of PLA signals (red dots) is reported in the graphs. Statistical significance ($p<0.01$) is indicated.
